# Obituary

**Published:** 1863-12

**Authors:** 


DIED—At Piqua, O., Nov. 19th, 1863, FRED. B. WHITE, dental student to Drs. Brown & Dills—aged 27 years.

I will not presume to write, here, the obituary of Frederick B. White, due one older in the profession, or due himself in another place; but I will be permitted to write of him as of one promising much for the profession as a type of a desirable class of dental students, and as a companion of one who feels his loss too deeply to refrain from any expression possible of his noble life, or lamented death.

Mr. White will be remembered by those connected with the Ohio Dental College during the years 1856, 1857 and 1858, as a friend and room-mate of the writer,—of tall, unusually commanding figure, combined with an affability irresistible in its tendency to form the acquaintance and friendship of all with whom he came in contact. A son of the late Dr. White—for twenty years President of the Wabash College, Crawfordsville, Ind. He enjoyed the advantages of a collegiate education, as well as those of a high social association. These general advantages were fully improved by the individual disposition. Never was culture bestowed upon more fruitful ground, nor labor more successfully spent in the formation of character; never was a young man's character more steadfast and elevated. This was his characteristic,—abounding and unyielding in habits of virtue and religion. Many and many number the nights I have seen him retire, yet never one without reading his Bible. And such habits were pleasingly blended with those natural to a highly social life. A serious invalid for four years prior to his death, most remarkably did he mingle, as it were, life and death,—walking hand in hand with his sable antagonist, as with an acquaintance neither dreaded nor desired. Ever noble and good, he was now afflicted, yet patient; suffering, yet resigned—perfectly resigned—approaching his grave

> "Like one that draws the drapery of his couch
> About him, and lies down to pleasant dreams."

But Oh! how hard for the living to resign "dust to dust"—the once noble breathing friend, to cold inanimate clay! no more to hear the accustomed footfall: no more to see the familiar face and gesture; or receive the greetings of his friendship, or the deeds of his kindness. Ah! no more is it for the accustomed members of our dental laboratory to gather round a common work-table, and make the sweat of the brow pleasant by association, and the laugh merrier by the many, and the sigh softer by the mutual sympathy!

Yet the bereavement of a few, by a sacred tie severed, is not to be obtruded on the many, only so far as it may remind us all, that

> "In the midst of life we are in death;"

and as we would appreciate life, and prepare for death, we would study, cultivate, and discharge our duties to the living—*worshiping the relationships of life*—as affording our highest duties, and purest joys. C. C. DALLS.

*Piqua*, O., Dec. 9, 1863.

